# Clinical Cases

**Published:** 1895-11

**Authors:** Robert T. Morris, P. H. Cronin


For Texas Medical Journal.

### CLINICAL CASES.

### SUPRAPUBIC CYSTOTOMY.

THE ORIGIN of this operation is properly due to Pierro Franco, performed by him about 1560, for stone, and by Wister, in 1818, for permanent drainage for an enlarged prostate, but it has never met with very general favor until Petersen introduced the rectal bag in 1880, which so modified the technique and removed the valid objections that it is now one of the safest and most satisfactory operations performed. In chronic cystitis, when non-operative measures fail, it is the ideal operation, and to Dr. Hunter McGuire is due the credit of placing it upon a permanent basis.

Its advantages over the perineal route are the following, viz.: It is a safer operation; it permits of inspection and better digital examination, and it does not cause so much discomfort and inconvenience to the patient. It may be performed at one seance, or two sittings; in the latter you cut to the bladder wall, pack with iodoform gauze, and then open the viscus a few days later.

A simple and rapid way of performing the operation is by introducing a large trocar, and then a Nelaton catheter, which will in some cases satisfactorily drain the bladder.

The following case will illustrate the technique usually observed:

C. S., age 24, presented himself for treatment June 16, with the following history: He had suffered with frequent micturition, eight to twenty times per day, for three years; had never had gonorrhœa or other venereal disease. An examination revealed a contracted meatus and urethral stricture in the spongy portion, and the urine showed an abundance of pus and mucus. The stricture would only permit a 9 American sound.

On July 14, the strictures were divided with Otis's urethretome to the size of 20 American. Salol had been given internally, and the bladder had been washed three times a week during the previous month, with nitrate of silver solution, one-half grain to the ounce, which was continued to July 29, when suprapubic cystotomy was performed. There was no noticeable improvement from cutting the strictures or from the bladder irrigation.

Operation.—The bladder was first washed with a saturated solution of boracic acid, after which the rectal bag was introduced and dilated with twelve ounces of water. Eight ounces of boracic acid solution was used to distend the bladder; then a three inch incision was made in the median line upward from over the symphysis pubis; the incision was carried down between the recti and pyramidalis muscles into the prevesical space. With as little disturbance as possible to the prevesical tissue, the bladder, which was in view, was caught with a tenaculum, and an inch incision made into its walls. The bladder incision was kept patent with two sutures, one on each side of the abdominal wound, enclosing the bladder wall, muscles and cutaneous tissues. Drainage was obtained by siphon action with a Nelaton catheter, which was held in place by a cutaneous suture. Some recommend two tubes, as affording better drainage, but I had no cause to complain of the single tube.

The bladder was washed three times daily, through the tube, with a saturated solution of boracic acid, for one month, when the tube was removed, and in a few days the opening closed.

The bladder symptoms subsided, and patient was on his feet, when orchitis in right testicle developed, necessitating a return to his bed and punctures of the tunica albuginea.

While a positive assertion as to the patient's continued improvement may be premature, his present condition is very satisfactory. He is on a fishing trip, and his bladder causes him no trouble.

The operation is devoid of danger, if due care is taken not to wound the peritoneum and unnecessarily disturb the prevesical tissue. There is no danger of rupturing a normal rectum with a twelve ounce dilatation.

I was kindly assisted in the operation by Drs. Scott, Cronin and Norsworthy.

R. T. MORRIS, M. D.

---

### ABDOMINAL SECTION.

In August, 1894, Mrs. M., age 42, called at my office, complaining of pain in right side and back of pelvis. She gave a history of gonorrhœa, and a previous serious illness, accompanied by a tumor on the right side, the contents of which, she said, was discharged through the uterus, after which she regained moderate good health. On examination, I found retroflected uterus, small tumor on right side continuous with cornea of uterus, quite an abundant leucorrhœa, uterus much enlarged and hyperemic; the uterus was firmly bound to posterior pelvis and rectum. I promised her possible relief from curettment, which operation was performed in October, 1894, the posterior vaginal pouch being packed on alternate days for a perion of six months. The curettment gave temporary relief, but two months after operation the discharge was nearly as bad as ever. The tampons relieved the pains in the back some, but the uterus remained as firmly bound as before. She was told that no further relief could be given her in the manner of treatment just described, and that her only hope for complete recovery was by the removal of her right tube, and attachment of the uterus to the abdominal walls.

She accepted the operation, and on August 1, 1895, assisted by Drs. Morris, Hathcock, Norsworthy and Scott, a section of the abdomen was made three inches long, through which the right ovary and tube was brought. The tube was found much thickened, and of large caliber; the ovary was the seat of many cysts, filled with a dark looking fluid. This tube and ovary was removed, and the left adnexia was then examined, and while the tube seemed in perfect condition, the ovary was as on the right side, cystic. We removed them also. About twenty fibrous bands held the uterus in its backward displacement. These were torn away, and the uterus anchored to the abdominal wall. After this procedure, hemorrhage of an alarming nature set in. Hot water was poured into the cavity for ten minutes, without avail. The Douglas pouch was then packed with sponges, and allowed to remain five minutes, with but slight decrease in the amount of hemorrhage. There was but two modes open to stop bleeding, viz.: to tampon with gauze, or enlarge opening and secure vessels. The former was practiced, the gauze being packed tightly behind the uterus, and allowed to pass through abdominal opening. Wire sutures were passed to close wound, and the final dressings applied. The gauze was allowed to remain in place for thirty hours, as a slight continual oozing of blood was in progress up to that time. On removal of gauze, the cavity was found to be walled off on every side by exudate. The wire sutures were then twisted, and abdominal wound closed, without drainage. On the fourth day temperature rose to 102° F., and small vessicles formed over abdomen and thighs; pulse 130; decided symptoms of septicæmia. Patient was put on quinine and abdominal wound dressed twice daily, and as symptoms subsided thought trouble was from stitch abscess. However, on twelfth day temperature again rose

102° F., pulse 140, severe pain in abdomen. A diligent search was made for the cause, by palpation and percussion, without finding any. That evening I was called in haste, to find the lower portion of the adominal wound open, and a foul smelling pus oozing through the opening. I at once enlarged same, put in drainage tubes, and washed out cavity, which I found to extend downward and forward between uterus and bladder. The cavity filled in nicely, and I had no further trouble, except a slight cystitis, which was relieved by washing the bladder every second day with a warm permanganate solution. My patient called at my office to-day, and says she has gained from four to five pounds in weight each week since I last dressed her wound, which was three weeks since, all pain having entirely disappeared.

I would suggest a few changes in the management of a similar case, i. e., where it is necessary to pack, a counter-opening should be made per vaginum, and the gauze removed by that rout, the abdominal wound being closed at once. Where fibrous bands cause the adhesion, they should under no circumstances be torn from either attachment, but divided through their center, if it be necessary to use the knife or scissors to accomplish this end. I feel sure the trouble I had was due to non-attention to the last mentioned precaution.

P. H. CRONIN, M. D.

